# Metabolic disorders in polycystic ovary syndrome: from gut microbiota biodiversity to clinical intervention

**DOI:** 10.3389/fendo.2025.1526468

**Published:** 2025-04-28

**Authors:** Jiyuan Li, Jiashen Qiao, Yihan Li, Gaofeng Qin, Yingjiang Xu, Kaixue Lao, Yanlin Wang, Yang Fan, Peiyi Tang, Lei Han

**Affiliations:** ^1^ The First School of Clinical Medicine, Binzhou Medical University Hospital, Binzhou Medical University, Binzhou, Shandong, China; ^2^ Department of Traditional Chinese Medicine, Binzhou Medical University Hospital, Binzhou, Shandong, China; ^3^ Department of Interventional Vascular Surgery, Binzhou Medical University Hospital, Binzhou, Shandong, China; ^4^ Department of Reproductive Medicine, Binzhou Medical University Hospital, Binzhou, Shandong, China; ^5^ Department of Clinical Nutrition, Chongqing University Jiangjin Hospital, Chongqing, China

**Keywords:** polycystic ovary syndrome, gut microbiota, metabolic disorders, insulin resistance, bile acids

## Abstract

Polycystic ovary syndrome (PCOS) is a prevalent gynecologic endocrine disorder characterized by menstrual irregularities, elevated androgen levels, and ovulatory dysfunction. Its etiology is multifactorial. Emerging evidence indicates that PCOS patients exhibit diminished gut microbiota (GM) diversity and altered microbial ratios, contributing to the metabolic derangements observed in these individuals. This review elucidates the role of GM in the pathogenesis and metabolic disorders of PCOS, encompassing insulin resistance (IR), hormonal imbalances, bile acid metabolic disorders, Interleukin-22-mediated immune dysregulation, and brain-gut axis disturbances. Additionally, it synthesizes current therapeutic strategies targeting the GM, aiming to furnish a theoretical framework for prospective clinical interventions.

## 1 Introduction

Polycystic ovary syndrome (PCOS) is a prevalent gynecological endocrine disorder with unclear etiology and pathogenesis. Increasing evidence suggests a significant association between human microbiota and the disease’s onset. The primary clinical manifestations of PCOS encompass elevated androgen levels, irregular menstruation, ovulatory disturbances, hirsutism, acne, and in some cases, obesity and insulin resistance (IR) (1). According to the 2023 International Evidence-Based Guidelines for the Evaluation and Management of PCOS in adults, diagnosis requires the presence of at least two of the following criteria after ruling out differential diagnoses: clinical or biochemical hyperandrogenism, ovulatory dysfunction, and polycystic ovary morphology confirmed by gynecologic ultrasonography or elevated serum Anti-Mullerian hormone levels (2). Notably, the guidelines recommend using serum AMH levels as an alternative to pelvic ultrasound for diagnosing PCOS in adults.

The human body harbors hundreds of billions of microorganisms in various parts, such as the oral cavity, intestinal tract, respiratory tract, and reproductive tract. These microorganisms possess significant metabolic regulatory and immune defense functions that impact the onset and progression of diseases. Particularly for women, the gut microbiota (GM) and the microbiota of the reproductive tract—including both the upper and lower sections—are crucial in regulating reproductive health. Numerous studies have demonstrated their association with the development of conditions like bacterial vaginosis, PCOS, endometriosis, and cervical cancer (3). Among these, the influence of GM on PCOS has been more extensively studied. This paper primarily reviews the alterations in gut microecology observed in PCOS patients, explores potential mechanisms linking GM to PCOS pathogenesis, and discusses therapeutic approaches based on GM.

PCOS is a complex condition influenced by both environmental and genetic factors. Poor dietary habits, sedentary lifestyles, diminished quality of life, genetic mutations, and defects can all contribute to the onset and pathology of PCOS. In conducting research, it is crucial to consider external variables that may impact GM composition and the pathophysiology of PCOS. Diets characterized by high fat and calorie content are more likely to induce disturbances in GM, whereas high-fiber diets and the Mediterranean diet—known for their nutritional balance and health benefits—often mitigate the incidence of PCOS and can be utilized in its treatment regimen (4). Adequate daily physical activity and a high quality of life can decrease the likelihood of obesity and IR. From a genetic standpoint, numerous candidate genes are implicated in the etiology of PCOS; alterations in these genes result in changes to metabolic pathways, thereby facilitating the progression of PCOS and ovarian dysfunction (5). However, it is essential to acknowledge the intricate nature of PCOS pathogenesis. Currently, there is a paucity of cohort studies investigating various factors, and existing studies are susceptible to confounding variables, resulting in considerable discrepancies among findings. Consequently, our research predominantly examines the relationship between GM and PCOS in the majority of patients. Further investigative efforts are warranted to elucidate how disparate environmental and genetic factors influence GM and PCOS.

## 2 Main functions and characteristics of GM in patients with PCOS

The human gut has a rich microbial community, comprising approximately 10^13 to 10^14 microorganisms. Among these microorganisms, some have already been classified and named (6). The human GM is mainly composed of five bacterial phyla comprise the main phyla: Firmicutes, Bacteroidetes, Proteobacteria, Actinobacteria, and Verrucomicrobia (7). The majority of these are Firmicutes and Bacteriodetes. Advances in research have revealed that GM plays a crucial role in food digestion, absorption, and maintaining systemic physiological balance (8). Based on their interaction with the host, the human microbiota performs essential functions that define and contribute to the physiology of the host, sharing a unique biological relationship termed a symbiosis. Emiley A et al. (9) discussed various symbiotic interactions between human hosts and microbiota in the context of maintaining bodily homeostasis, finding that microbes can have beneficial effects on the human body through complex mechanisms, while these interactions can also lead to dysbiosis. Beneficial bacteria, such as *lactobacilli* and *bifidobacteria*, can protect the intestinal mucosa and inhibit the growth of harmful bacteria, while harmful bacteria, which include *Staphylococcus*, *Salmonella*, and *Campylobacter*, produce toxins and damage the intestinal mucosa (10). Under normal conditions, the GM maintains a delicate dynamic balance, supported by the strong barrier function of the intestinal mucosa, which blocks bacterial invasion and detoxifies harmful substances. This protective mechanism operates through two primary processes: Firstly, the intestinal mucosa secretes copious amounts of mucus to shield itself from bacterial intrusion and neutralize toxins (11). Secondly, tight junctions composed of adhesion proteins facilitate selective absorption, preventing large molecules or bacteria from penetrating the intestinal walls and staving off various diseases (12).

The disruption of GM is linked to numerous diseases, including the pathogenesis of PCOS, while the occurrence of PCOS also affects the intestinal microecological balance to a certain extent (13). Studies have demonstrated alterations in the diversity and composition of intestinal microbiota in patients with PCOS. The α-diversity, which refers to the diversity within a single sample or group, indicates species abundance and variety within a particular community. Conversely, the β-diversity measures the similarities or differences between distinct samples or groups. Yu et al. observed a decreasing trend in the diversity of GM in PCOS mice (14), and studies in humans have reported reduced α-diversity and altered β-diversity in PCOS patients (15). However, findings on β-diversity are inconsistent across studies (16). Some research suggests that higher α-diversity of GM correlates with better host health, whereas lower values are linked to metabolic or endocrine disorders (17). However, we cannot simply assume that high alpha diversity is always beneficial. Regarding changes in the composition of GM, it has been found that women with PCOS exhibited a significant increase in Fusobacteria, Proteobacteria, and Bacteroidetes while showing a significant decrease in Tenericutes and Firmicutes compared to healthy controls at the phylum level (16). Changes have also been observed at the genus level, with an increase in *Bacteroides*, *Lactobacillus*, and *Escherichia/Shigella* and a decrease in *Barnesiella*, *Alloprevotella*, and *Coprococcus* (16). The elevation of pro-inflammatory bacteria, such as those within the *Bacteroidetes*, leads to a series of metabolic changes. Specifically, *Bacteroides* has been associated with promoting IR in women with PCOS (18). Liu et al. found that certain gram-negative bacteria belonging to *Bacteroides* and *Escherichia/Shigella* were significantly increased in the intestinal tracts of women with PCOS combined with obesity, but a decrease in *Akkermansia*, which has some protective effect on the intestinal mucosa (19). The levels of beneficial bacteria, such as *lactobacilli* and *bifidobacteria*, were significantly reduced in PCOS patients, which is not conducive to improving intestinal immunity. Due to the complexity of GM, the results of different studies are sometimes biased, controversial, and not yet fully understood.

Dietary habits exert a significant influence on GM. Research has indicated that patients with PCOS exhibit significantly lower dietary fiber intake compared to healthy individuals (20). As research in this area has evolved, it has been theorized that dietary fiber serves as an excellent substrate for biofilm formation. In the context of GM, biofilm formation promotes synergistic interactions both among bacteria and between bacteria and their host. For instance, lactic acid bacteria demonstrate heightened bioactivity when forming biofilms on substrates such as wheat bran (21). The inadequate dietary fiber consumption prevalent among PCOS patients can detrimentally impact the composition and function of healthy GM. Dietary patterns in PCOS patients, often characterized by high fat and high energy content, frequently lead to obesity. Studies have identified an elevated Firmicutes-to-Bacteroidetes ratio at the phylum level as a distinctive feature of obese GM (22). Intestinal microbiota composition varies among PCOS patients from different geographical regions and with differing testosterone levels. Notably, *Alistipes* is predominantly enriched in European PCOS patients, while *Blautia* and *Roseburia* are more abundant in Chinese patients. Furthermore, substantial differences in GM have been observed between PCOS patients with higher testosterone levels and those with lower testosterone levels, suggesting that racial and regional factors also play a role in shaping GM (23).

Lastly, we have found that studies on individual patients’ GM may deviate somewhat from epidemiological statistical results. This discrepancy may be due to differences in research methods and existing errors in studies, necessitating a comprehensive consideration of changes in GM.

## 3 Possible mechanisms of GM dysregulation causing polycystic ovary syndrome

Alterations in GM induce a spectrum of metabolic disorders within the body, encompassing glucose metabolism irregularities characterized predominantly by IR, dysregulation of lipid metabolism associated with obesity and alterations in short-chain fatty acid (SCFAs) profiles, perturbations in sex hormone metabolism marked by elevated levels of luteinizing hormone (LH) and androgens, as well as disturbances in amino acid and bile acid metabolism, among others. These combined alterations in microbial composition and metabolic pathways contribute to the heterogeneous clinical presentation of PCOS. Conversely, the interplay between metabolic imbalances and inflammatory responses can further exacerbate GM dysbiosis, perpetuating a cycle of mutual exacerbation.

### 3.1 GM and IR

IR is a prevalent issue in patients with PCOS and is considered a core component of its pathogenicity. He et al. found that the GM composition of PCOS patients with IR differed from that of PCOS patients without IR and healthy women (24), suggesting a close relationship between disturbances in GM and IR.

When GM is disrupted by various factors such as diet, medication, and stress, the number of beneficial bacteria decreases while that of harmful bacteria increases. This imbalance leads to an increase in harmful substances such as lipopolysaccharide (LPS) and heightened activity of pro-inflammatory factors. These changes weaken the tightly connected barrier function of the colonic epithelium, resulting in increased permeability of the intestinal wall. Consequently, there is an elevated transfer of endotoxin to the mucosal epithelium and its entry into the bloodstream through the compromised intestinal epithelium, which affects various organs and systems, contributing to the development of “leaky gut” syndrome (25). Some researchers propose that the entry of LPS into the bloodstream due to a leaky gut may activate the immune system, which in turn impairs the function of insulin receptors, leading to reduced insulin efficacy, so the application of antibiotics to inhibit Gram-negative bacilli and reduce LPS entry into the bloodstream in animal models has been shown to improve insulin sensitivity (26). This is known as the Dysbiosis of GM theory of PCOS. Studies have also demonstrated that direct intravenous injection of LPS into the somatic circulation of mice and humans results in elevated fasting blood glucose (FBG) and insulin levels, however, oral administration of the beneficial bacterium Lactobacillus acidophilus followed by LPS injection keeps normal insulin sensitivity (27). Furthermore, LPS induces a systemic inflammatory response, promotes the expression of inflammatory cytokines such as tumor necrosis factor-alpha (TNF-α) and interleukin-6, exacerbates inflammation, interferes with insulin receptor function, and reduces insulin sensitivity, manifesting as IR (28).

SCFAs such as acetic acid, propionic acid, and butyric acid are volatile compounds produced by intestinal microorganisms during the fermentation of dietary fibers. These SCFAs play a crucial role in regulating glucose and lipid metabolism. In pancreatic β-cells, SCFAs interact with glucose-insulin secretion via the FFAR2 and FFAR3 receptors, leading to the release of peptide hormones that regulate hunger and enhance insulin sensitivity (29). Patients with PCOS have significantly lower levels of SCFAs in their intestines compared to healthy controls (30), which may be associated with disturbances in GM. Oral administration of sodium butyrate has been shown to improve insulin sensitivity in diabetic mice, but its effect on IR in PCOS patients remains unexplored (31). Additionally, SCFAs inhibit inflammation and regulate immune homeostasis (32). Butyric acid, in particular, can mitigate LPS-induced apoptosis and oxidative stress while enhancing glucose metabolism in human ovarian granulosa tumor cells under inflammatory conditions (33). Furthermore, SCFAs enhance the barrier function of the intestinal mucosa and reduce the occurrence of “leaky gut” syndrome (26).

Qiao Jie and colleagues introduced the novel concept of intestinal bacterial-derived host isoenzymes, demonstrating that during the coevolution of intestinal microbiota and their hosts, certain bacteria produce enzymes that mirror those of the host and contribute to disease processes. A notable example is Dipeptidyl peptidase 4, predominantly synthesized by members of the Bacteroidetes phylum. In scenarios where the intestinal barrier integrity is compromised, microbial-derived DPP4 can disrupt glucose homeostasis by inactivating glucagon-like peptide-1, akin to the function of its human counterpart (34).

Branch-chain amino acids (BCAAs), including valine, leucine, and isoleucine, are crucial nutritional signals and metabolic regulators in the body, playing a significant role in glucose homeostasis. Glucose and amino acid metabolism are closely interlinked. In patients with PCOS, the catabolism and synthesis of BCAAs are abnormal. Elevated levels of BCAAs in the blood may negatively impact the development of IR, glucose intolerance, type 2 diabetes, and obesity (35). Some researchers have suggested that plasma levels of BCAAs could serve as predictors for the onset of type 2 diabetes (36). Studies have shown that serum levels of BCAAs are significantly elevated in IR individuals, presumably due to the involvement of GM in the metabolism of BCAAs (37). Furthermore, in other studies, treatment with BCAAs has been found to mitigate the effects of high-fat diet-induced metabolic fatty liver disease through a mechanism involving gut microbes (38), although the specific mechanism of action remains unclear.

### 3.2 GM and hormone metabolism disorders

Hyperandrogenemia (HA) is also a pivotal clinical manifestation in patients with PCOS, potentially stemming from complex interplays involving the Hypothalamic-pituitary-gonadal (HPG) axis, adrenal function abnormalities, dysregulation of glucose and lipid metabolism, intestinal microbiota imbalances, chronic inflammation, and genetic predispositions (39). Comparative analysis of fecal metabolites between PCOS patients and healthy controls revealed marked disparities, suggesting that reduced alpha diversity within GM correlates with elevated total testosterone levels and hirsutism in PCOS individuals, implying a regulatory role of GM on circulating testosterone concentrations. Conversely, evidence indicates that androgens may modulate GM composition in female rats (40). Specifically, the abundance of *Alloprevotella* positively correlates with androgen levels, while *Candleria* abundance is associated with circulating androstenedione concentrations. Probiotic interventions in PCOS patients have demonstrated efficacy in mitigating serum testosterone levels and alleviating symptoms such as hirsutism.

**Figure 1:**
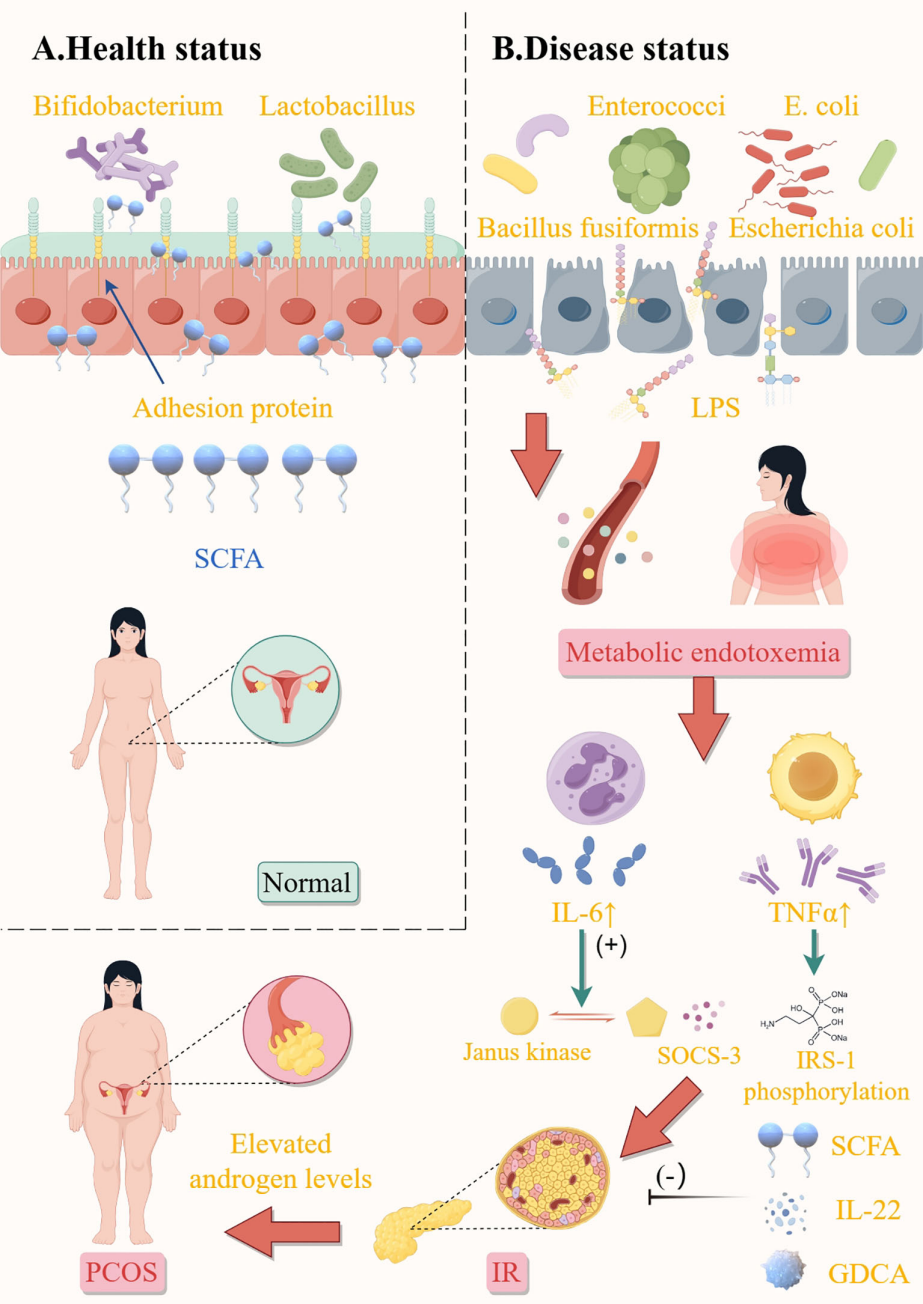
Influence of gut microbiota on elevated androgen levels in patients with PCOS. Health status **(A)**. Balanced gut microbiota produce sufficient short-chain fatty acids (SCFAs) to strengthen the intestinal barrier, suppress inflammation, and enhance insulin sensitivity; protective mechanisms prevent LPS leakage and maintain metabolic homeostasis. Disease status **(B)**. Harmful bacteria promote LPS release, lead to endotoxemia, and promote the expression of tumor necrosis factor-α (TNF-α), interleukin-6, and other inflammatory factors, then cause insulin resistance and PCOS-related symptoms. In addition, SCFA, IL-22, and GDCA inhibited the development of insulin resistance. *E.coli, escherichia coli;* SCFA, short-chain fatty acids; LPS, lipopolysaccharide; IL-6, interleukin-6; TNFα, tumor Necrosis Factor-alpha; SOCS-3, suppressor of cytokine signaling 3; IRS-1, insulin receptor substrate 1; IL-22, interleukin-22; GDCA, glycodeoxycholic acid; IR, insulin resistance; PCOS, polycystic ovary syndrome. This figure is made by Figdraw.

The intricate mechanisms underlying the interaction between sex hormones and GM remain elusive. Tremellen et al. previously introduced the Dysbiosis of GM theory to elucidate PCOS symptomatology, postulating that dietary-induced GM imbalances heighten intestinal mucosal permeability, facilitating increased *Escherichia coli* influx and elevating circulating LPS levels (26). This immune activation disrupts insulin receptor functionality and elevates serum insulin, subsequently augmenting ovarian androgen production and impairing follicular maturation (**Figure 1**). The research underscores that aberrant androgen and estrogen synthesis in PCOS predominantly originates from the follicular membrane and granulosa cells, with a contributory role from the adrenal cortex. Overexpression of cytochrome P450 17α-hydroxylase A1 in PCOS follicular membrane cells fosters excessive androgen accumulation and endogenous steroidogenesis disruption. Conversely, downregulated CYP19A1 expression impedes androgen-to-estrogen conversion. Regulation of androgen synthesis affects follicular development and ovarian function through multiple pathways: Androgen-promoting pathways: IRS-PI3K/cAMP/PKA, MAPK/ERK (via FGF13/LH/CYP17), NF-κB (via TNF-α activation/CYP17 up-regulation), and miR-130b-3p (targeting MAPK1/PI3K) all promote androgen production. BMP4-Smad inhibition is blocked by the androgen receptor, further exacerbating androgen accumulation. Anti-androgen pathway: LKB1-IGF inhibits androgen and promotes estrogen by down-regulating CYP17A1 and up-regulating CYP19A1. Downregulation of CYP19A1 by AKAP95-cAMP/PKA and melatonin-ERK pathways hindered the conversion of androgen to estrogen, resulting in imbalance. Pathogenesis of hyperandrogenism: DHT inhibited granulosa cell proliferation through AMPK/ERK (P27 kip). RNF6 inhibits GDF9-induced follicle arrest by ubiquitination of AR. PI3K-AQP9 reduces water transport function. Abnormal metabolism: FBP1 is related to insulin/glucose metabolism disorder; Decreased MAPK/ERK activity leads to mitochondrial apoptosis. PTEN-PI3K/Akt regulates the proliferation and differentiation of granulosa cells. The interaction of multiple pathways leads to follicular abnormality and polycystic ovarian pathology, reflecting the complexity of the hormone-molecular network (41). Notably, *Escherichia coli* supplementation has been shown to enhance Interleukin-22 (IL-22) production in granulosa cells, mitigate mitochondrial damage in PCOS mice through sex hormone restoration and ovarian tissue morphology improvement, suppress deleterious GM populations, and facilitate aminoglycan and nucleotide sugar metabolic pathways (42).

Furthermore, HA and IR share a reciprocal relationship, exacerbating each other into a self-perpetuating cycle: hyperinsulinemia secondary to IR amplifies free testosterone by diminishing sex hormone-binding globulin. Conversely, HA influences gut microbial community structure, modifying intestinal permeability and initiating the IR cascade. Additionally, augmented androgen secretion promotes visceral fat catabolism, elevating fatty acids and intensifying IR, thereby fueling PCOS progression (3).

### 3.3 GM and bile acid metabolism disorder

Bile acids divided into free and bound bile acids by structure, primary and secondary bile acids by source, hydrophilic and hydrophobic bile acids by molecular group. Hydrophilic bile acids have a protective effect on liver cells, whereas hydrophobic bile acids are cytotoxic. Excessive accumulation of these acids can result in hepatocyte damage, necrosis, and apoptosis.

Bile acids primarily regulate the body’s metabolism by binding to specific receptors. Common receptors include the farnesoid X receptor (FXR) and G protein-coupled receptors (43). Among these, goose deoxycholic acid is a potent activator of the farnesoid receptor (44). Secondary bile acids exhibit a stronger affinity for G protein-coupled receptors. FXR is abundantly expressed in intestinal epithelial cells, hepatocytes, and intestinal endothelial cells, and its activation capacity correlates with the structure of bile acids (hydrophobicity) (45). There are significant differences in GM of PCOS patients compared to healthy individuals. Specific gut bacteria (such as *Ruminococcus*, *Lachnospiraceae*, and *Prevotella*) are less abundant in PCOS patients, while the abundance of *Lactobacilli*, *Streptococcus*, and *Escherichia coli* is higher (46). This dysbiosis may affect bile acid metabolism, thereby affecting the activation of FXR.

Relationship between bile acid metabolism and the FXR pathway in polycystic patients: (1) In hepatocytes, activation of FXR induces the small intestine-specific transcription factor SHP to inhibit the transcription of CYP7A1 and CYP8B1 genes, thereby reducing bile acid synthesis. Polycystic patients may have metabolic disorders that lead to abnormal synthesis and metabolism of bile acids, thereby affecting the function of FXR (16). (2) FXR can also promote bile acid secretion by inducing the bile salt export pump (BSEP) in the liver and inhibiting the expression of NTCP (sodium-taurocholate cotransporting polypeptide), reducing the reabsorption of bile acids from the blood back into the liver. This regulation may be affected in polycystic patients, leading to abnormal circulation and metabolism of bile acids. (3) FXR activity may affect lipid metabolism and endocrine function in polycystic patients (15). Since FXR can regulate bile acid synthesis and transport, impaired function may lead to abnormal accumulation or deficiency of bile acids, thereby affecting lipid metabolism and insulin sensitivity. Additionally, FXR indirectly affects bile acid metabolism by regulating the composition and function of GM. Changes in GM may further affect the FXR signaling pathway, forming a complex feedback mechanism. In summary, the FXR pathway may affect the metabolism and endocrine function of polycystic patients by regulating bile acid synthesis, transport, and the composition of the microbiota.

Takeda G-protein-coupled receptor (TGR5) receptor is widely expressed in the heart, skeletal muscle, liver, small intestine, and peripheral blood leukocytes, among other tissues. It is activated by bile acids with varying affinities. Since TGR5 is expressed in intestinal neurons, a reduction or deficiency of TGR5 receptors can disrupt intestinal epithelial tight junctions, thereby affecting intestinal motility (47). As a membrane-bound receptor for bile acids, TGR5 also plays significant roles in glycolipid metabolism, anti-inflammation, and immunomodulation.

**Figure 2:**
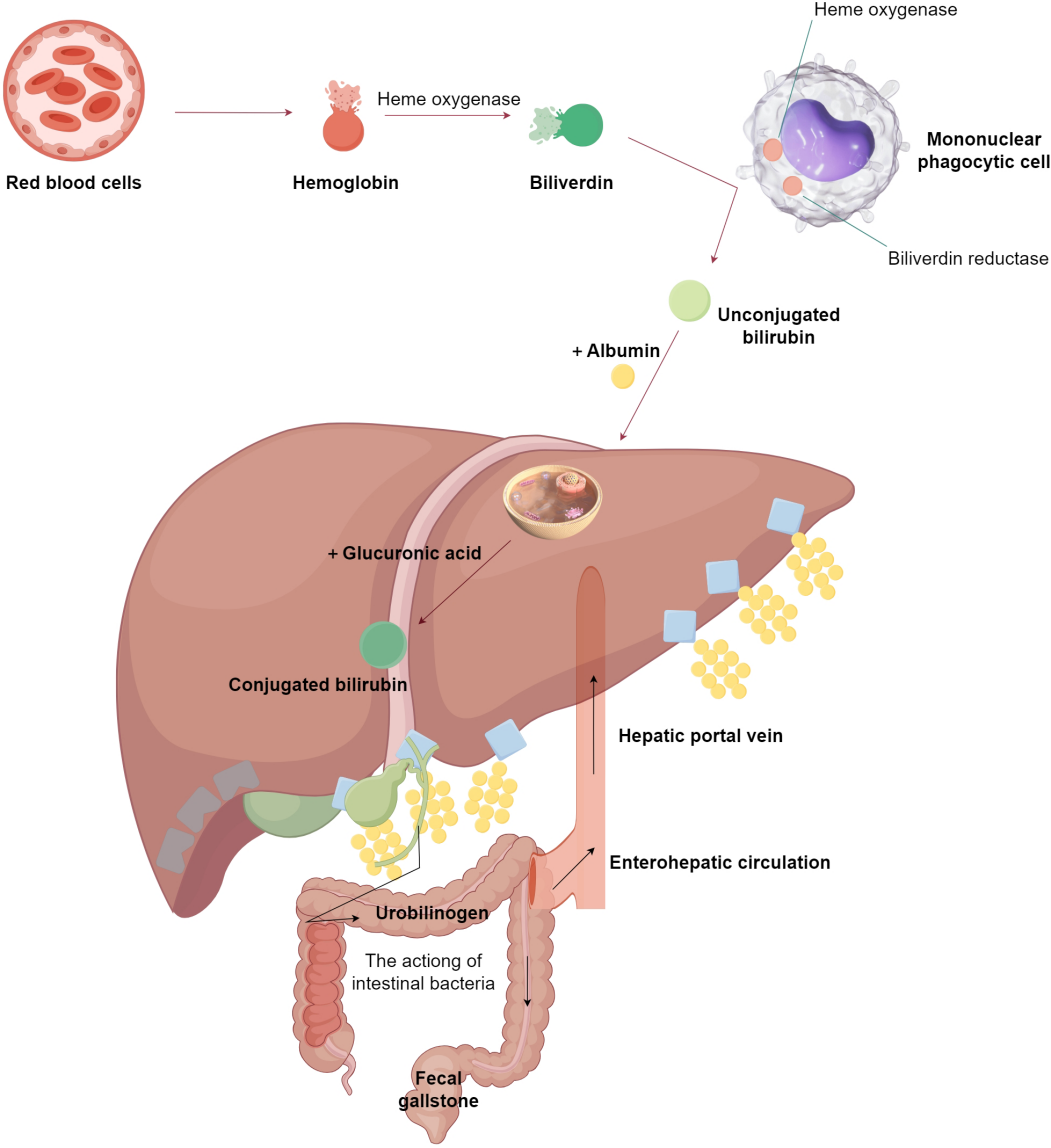
The normal metabolic process of bile acids. This figure is made by Figdraw. As shown in the figure, it primarily illustrates the metabolic pathway of bile acids in a normal organism, which requires the synergistic actions of the liver, intestines, and gut microbiota.

The metabolic pathway of bile acids, encompassing bile production, conversion, excretion, and reabsorption, constitutes the enterohepatic cycle of bile acids. This pathway necessitates the interplay of hepatic, intestinal, and GM functions. Cholesterol is transformed in the liver to produce primary free bile acids. Within the hepatocyte, these primary free bile acids are condensed to form primary conjugated bile acids, which are then concentrated by the gallbladder and released into the intestines. Here, they undergo reduction and deoxygenation to generate secondary free bile acids, facilitated by intestinal bacteria. These secondary free bile acids are reabsorbed into the liver, where they are conjugated with glycine or taurine to form secondary conjugated bile acids. Following the consumption of fatty foods, the digested and absorbed chyme can stimulate the secretion of pancreatic enzymes, which in turn prompts bile duct cells to secrete bicarbonate and water, increasing the content of bile; cholecystokinin stimulates the gallbladder to contract, thereby discharging bile into the duodenum to aid in the digestion and absorption of lipids. Approximately 95% of bile acids are reabsorbed and enter the liver via the portal vein, where free bile acids are conjugated and re-enter the intestine with bile, completing the enterohepatic circulation of bile acids (48). Bile acids that do not participate in this cycle are biotransformed by intestinal bacteria in the colon to produce secondary bile acids, such as deoxycholic acid and lithocholic acid (49). The enterohepatic circulation of bile acids plays a role in the digestion and absorption of fatty foods, promotes the excretion of bile acids, and inhibits the precipitation of cholesterol in bile (**Figure 2**).

**Figure 3:**
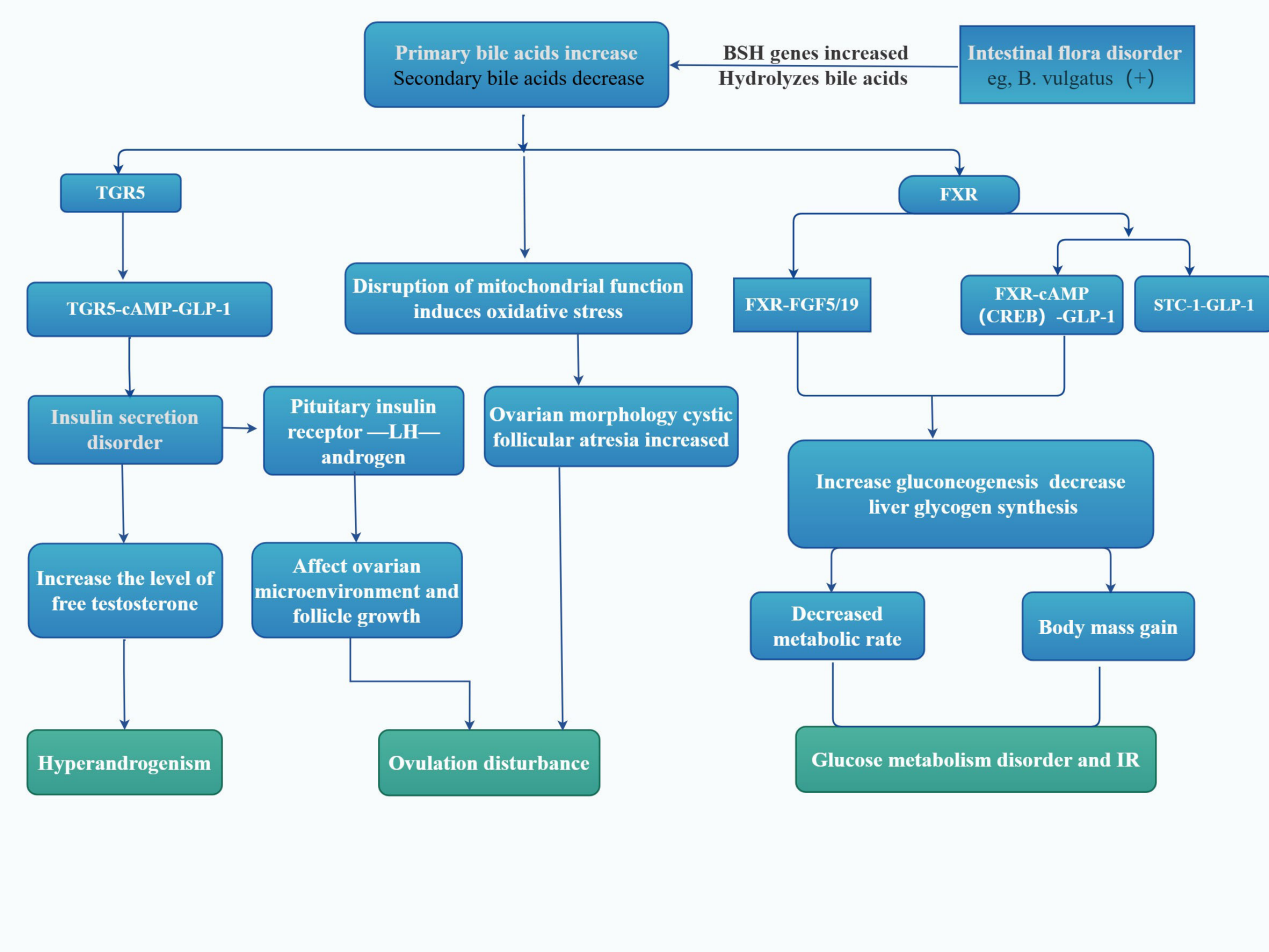
Microbial regulation of the PCOS and bile acid receptors. As shown in the figure, by linking the impact of intestinal microbes on bile acid metabolism, it describes the roles of bile acid metabolic FXR and TGR5 microbial regulation on the body of patients with PCOS. FXR, farnesol receptor; TGR5, takeda G-protein-coupled receptor 5; cAMP, cyclic adenosine monophosphate; GLP-1, glucagon like peptide-1; FGF19, fibroblast growth factor 19; STC-1, stanniocalcin-1; LH, Luteinizing hormone; BSH, bile salt hydrolase; IR, Insulin resistance; This figure is made by Figdraw.

In the intestine, GM deconjugates conjugated primary bile acids through bile salt hydrolase and demethylates them via 7α-dehydroxylase, for example (**Figure 3**), converting cholic acid into deoxycholic acid and chenodeoxycholic acid (CDCA) into lithocholic acid. These transformation processes involve the action of various bacterial enzymes, such as *BaiA*, *BaiB*, and *BaiE*, among others. *Bacteroides vulgatus*, a common type of Bacteroides, is significantly upregulated in GM of women with PCOS. It can deconjugate conjugated bile acids synthesized by the liver, leading to a significant increase in the abundance of bile salt hydrolase genes. This subsequently affects the metabolism of these hosts’ glycodeoxycholic acid (GDCA) and taurodeoxycholic acid, resulting in significantly reduced levels of GDCA and taurodeoxycholic acid in both feces and serum (50).

Additionally, GM can regulate hepatic bile acid synthesis. It has been observed that GM in conventionally reared mice suppresses bile acid synthesis compared to germ-free mice. This suppression may be attributed to the reduced activity and gene expression of cholesterol 7a hydroxylase, a key enzyme for CDCA synthesis, in conventionally fed mice, which results in a decrease in the level of muriatic acid produced by hydroxylation of CDCA in liver microsomes. Therefore, alterations in GM can impact bile acid metabolism by engaging receptors or directly affecting bile acid synthesis, leading to disorders in bile acid metabolism (**Figure 3**).

The impact of bile acid changes in patients with PCOS on the body is primarily reflected in the following aspects. Firstly, there is a close relationship between bile acid changes and GM. Significant differences exist in the GM of PCOS patients compared to healthy individuals, with specific gut bacteria such as Lactobacilli, Streptococcus, and Escherichia coli being more abundant in PCOS patients, while Ruminococcus, Lachnospiraceae, and Prevotella are less abundant. This imbalance in GM may lead to alterations in bile acid metabolism and synthesis, resulting in elevated levels of major conjugated bile acids such as GCA, TCA, and GCDCA in PCOS patients, thereby affecting ovarian function and causing ovulatory disorders. Secondly, inflammation and IR are also significant consequences of bile acid changes. Endotoxins produced by GM, such as LPS, enter the bloodstream and activate TLR4 receptors, leading to increased expression of TNF-α and interleukin-6, which in turn induce IR and inflammatory responses. These reactions may promote the occurrence and development of PCOS. Furthermore, bile acid changes are closely related to immune responses. Changes in bile acids may affect the secretory function of intestinal type 3 innate lymphoid cells, thereby regulating the secretion of IL-22, which plays a crucial role in modulating intestinal immunity and inflammation. An imbalance in bile acids may lead to alterations in these immune responses, thereby influencing the pathological process of PCOS. In summary, bile acids may play a significant role in the formation and development of PCOS by regulating GM, affecting ovarian cell function, inducing inflammatory responses, and modulating immune responses through various mechanisms.

### 3.4 GM and IL-22 pathway

IL-22 is a molecule secreted by intestinal innate lymphoid cells that exerts various protective effects and plays a crucial role in maintaining intestinal immune homeostasis (51, 52). IL-22 is involved in the regulation of a variety of chronic inflammatory diseases and, in numerous instances, diseases related to glucose metabolism abnormalities, such as hyperglycemia and IR, can be controlled and alleviated through the exogenous infusion of artificial IL-22 in obese mice.

A related study by Academician Jie Qiao demonstrated that changes in GM of patients with PCOS, specifically, an increased abundance of B. vulgatus, led to reduced levels of GDCA and tauro-ursodeoxycholic acid in feces and serum, as well as decreased levels of IL-22 in serum and follicular fluid (37). Bile acids regulate IL-22 production, and GDCA and tauro-ursodeoxycholic acid induce IL-22 secretion from intestinal type 3 innate lymphoid cells by promoting GATA3 expression. Infusion of IL-22 or GDCA into PCOS mice alleviated IR, promoted white fat browning, and inhibited inflammation of follicular granulosa cells, thereby reversing PCOS to some extent (37).

Thus, the disturbances in GM of PCOS patients are primarily characterized by an increased abundance of Bacteroides vulgatus and a substantial production of bile salt hydrolase genes, which encode bile salt hydrolases. This leads to disruptions in bile acid metabolism within the organism. The associated decrease in bile acid concentration results in a reduction of IL-22. Under normal conditions, IL-22 regulates metabolism via the STAT3 signaling pathway. Inhibition of this pathway attenuates the browning of white adipose tissue, thereby disrupting glucose-lipid metabolism and increasing IR. Furthermore, the inhibition of islet B cell regeneration and the promotion of apoptosis exacerbate the inflammatory response, leading to ovulatory dysfunction, polycystic ovarian changes, and localized inflammation of the ovary (1).

### 3.5 GM and the brain-gut axis

The brain-gut axis represents a bidirectional signaling network that connects the gastrointestinal tract with the central nervous system. Numerous studies have demonstrated the correlation between the gut and the brain, revealing that some of the same peptides exist in both the GI tract and the central nervous system. These peptides, including ghrelin, Peptide YY (PYY), and serotonin, are also known as brain-gut mediators. In women with PCOS, levels of serotonin, ghrelin, and PYY are significantly reduced in the blood compared to women without PCOS. It has been discovered that specific GM affect the secretion of intestinal hormones. For instance, *Bacteroides*, *Escherichia/Shigella*, and *Blautia* negatively correlate with ghrelin, whereas *Akkermansia* positively correlates with ghrelin (19). Furthermore, the proliferation of some bacteria that produce short-chain fatty acids, such as *Faecalibacterium prausnitzii*, *Butyricimonas*, and *Akkermansia*, leads to an increase in intestinal short-chain fatty acid production. These fatty acids can bind to receptors on the membranes of enteroendocrine cells and stimulate the direct release of gut-brain mediators, such as the gastric starvation hormone and PYY[9]. Consequently, changes in GM and short-chain fatty acids in PCOS patients can trigger the release of brain mediators, such as gastric starvation hormone and PYY, which in turn affect the secretion of sex hormones, the regulation of central neurotransmitters, and downstream neuronal functions as well as immunomodulation (53).

#### 3.5.1 Hypothalamic-pituitary-gonadal axis

Hypothalamic gonadotropin-releasing hormone (GnRH) neurons secrete GnRH, which regulates the secretion of follicle-stimulating hormone (FSH) and LH from the pituitary gland. Gonadotropins act on the gonads to regulate the secretion of sex hormones and reproductive function. Studies have shown that over-activation of GnRH neurons is pivotal in the development of PCOS (54).

**Figure 4:**
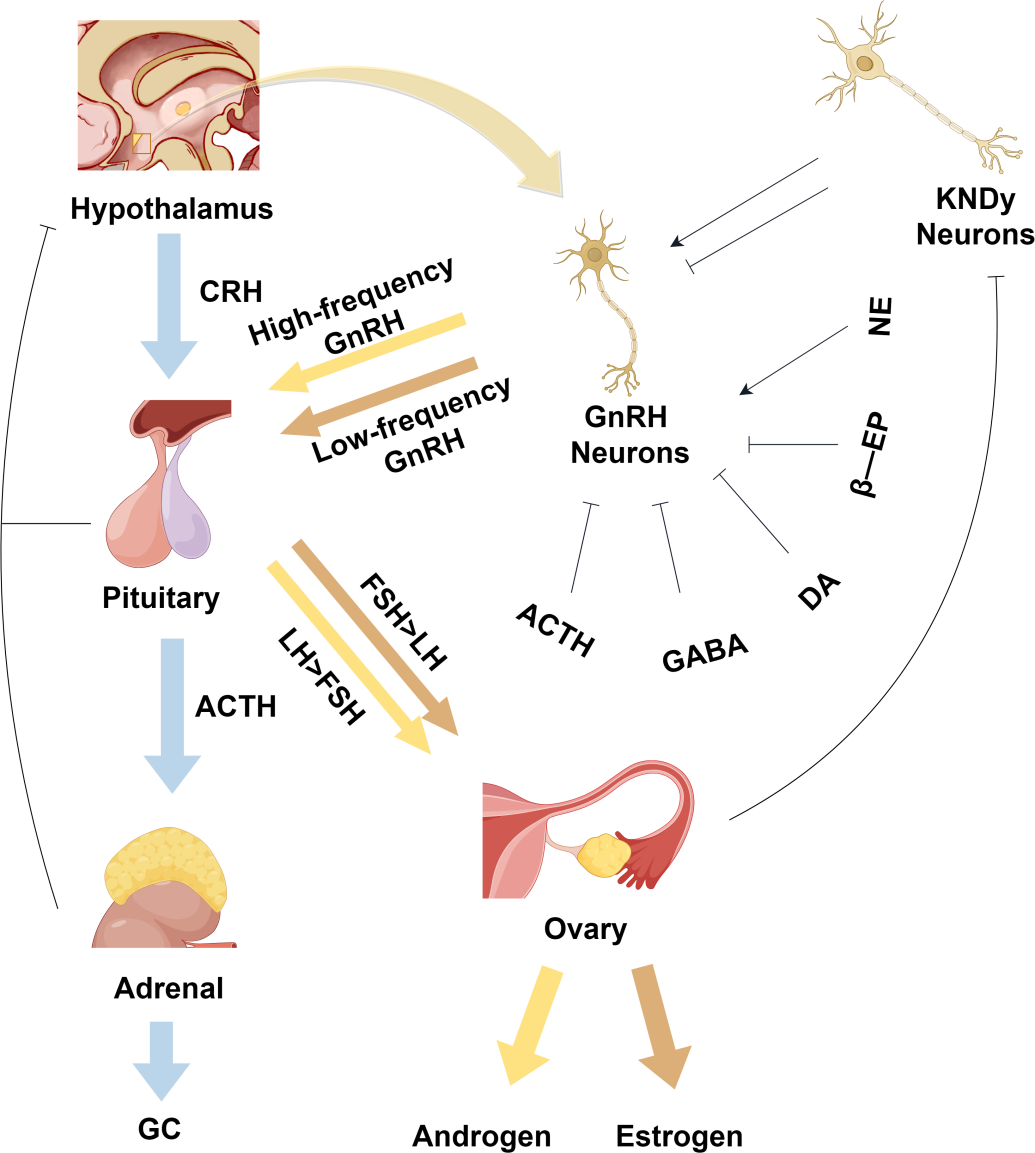
Physiological regulatory mechanisms of the normal hypothalamic-pituitary-hormonal axis. As shown in the figure, it depicts the HPG axis and the HPA axis, where KNDy neurons can regulate GnRH neurons. GnRH neurons generate high-frequency pulse signals to promote the release of FSH hormone, which in turn facilitates the release of estrogen. On the other hand, when GnRH neurons produce low-frequency pulse signals, it promotes the release of LH hormone, thereby facilitating the release of androgen. CRH, corticotropin-releasing hormone; ACTH, adrenocorticotropic hormone; GC, glucocorticoid hormones; GnRH, gonadotropin-releasing hormone; FSH, follicle-stimulating hormone; LH, luteinizing hormone; KNDy, kisspeptin/neurokinin B/dynorphin; GABA, gamma-aminobutyric acid; DA, dopamine; NE, norepinephrine; β-EP, beta-endorphin. This figure is made by Figdraw.

PCOS is characterized by an increase in GnRH production and secretion, which, through negative feedback, leads to a decrease in sex steroids at the hypothalamic level. Neurons in the infundibular nucleus of the hypothalamus release GnRH in a pulsatile manner, resulting in increased secretion of LH and FSH (High-frequency pulses promote LH secretion, while low-frequency promotes FSH secretion) (55). In women with PCOS, elevated levels of LH result in high androgen production by the ovarian follicular membrane, while a relative lack of FSH results in impaired follicular growth, polycystic ovarian morphology, and sporadic ovulation. Since GnRH neurons themselves do not have estrogen or progesterone receptors, the Kisspeptin/neurokinin B/dynorphin neurons upstream of them play a crucial role in the reduction of feedback from sex steroids on GnRH release. Kisspeptins promote GnRH release from GnRH neurons via the GPR54 receptor, which regulates the positive and negative feedback of estradiol (56). Gamma-aminobutyric acid (GABA) is primarily an inhibitory neurotransmitter. Due to the high concentration of intracellular chloride ions in GnRH neurons, GABA stimulates GnRH neurons through GABAA receptors and promotes the synthesis and release of GnRH from GnRH neurons. **Figure 4** summarizes the mechanism of action of the normal hypothalamic-pituitary-hormonal axis and its regulatory processes (**Figure 4**).

Currently, there is limited research on GM relationship with the HPG axis. Some studies suggest that GM may disrupt hormone secretion by affecting the HPG axis, but the specific mechanisms remain unclear. These mechanisms may be related to several microbial-derived molecules, such as SCFAs. The microbiota can also independently produce or promote the production of many neuroactive molecules, including but not limited to gamma-aminobutyric acid, serotonin, norepinephrine, and dopamine. However, it is still unclear whether these molecules reach the relevant receptors or attain sufficient levels to trigger host responses (53). Our article primarily summarizes the regulation and abnormal states of the HPG axis in patients with PCOS. Further investigation is needed to determine whether these abnormal states are associated with GM.

#### 3.5.2 Hypothalamic-pituitary-adrenal axis

When the human body is under stress, such as from overwork, tension, or life pressures, the HPA axis becomes activated. Under these conditions, the paraventricular nucleus of the hypothalamus secretes corticotropin-releasing hormone (CRH) and arginine vasopressin (57). CRH acts on the anterior pituitary gland to stimulate the secretion of adrenocorticotropic hormone (ACTH), which in turn acts on the adrenal cortex to induce the synthesis and release of glucocorticoid hormones (GC). GCs bind to the glucocorticoid receptor in the hippocampus, activate the negative feedback pathway of the HPA axis, and regulate the release of peptide hormones from the hypothalamus and pituitary gland to achieve a relatively stable state of the internal environment (**Figure 4**).

A substantial body of research has demonstrated that prolonged exposure to stress in patients with PCOS results in elevated cortisol levels. This, in turn, causes cortisol receptor insensitivity and further stimulates the HPA axis. Consequently, this leads to the disruption of cortisol’s negative feedback regulation and persistent activation of the HPA axis, ultimately contributing to the onset of various stress-related diseases, including depression. The pathogenesis of depression is linked to alterations in the HPA axis and reduced levels of monoamine neurotransmitters. Additionally, obesity, IR, hyperandrogenemia, inflammation, and infertility are all risk factors for PCOS and are believed to play a role in the development of depression (58, 59).

In patients with PCOS, the HPA axis is persistently activated, leading to hypersecretion of ACTH. This results in a decrease in the frequency and amplitude of GnRH pulses and a reduction in gonadotropin levels. Elevated ACTH levels, however, cause an increase in androgen synthesis, which, after certain transformations, also raises the amount of estrogen. These two hormones synergistically inhibit the sex function regulation axis of the pituitary gland through feedback mechanisms. Consequently, the disorders of the HPA axis and HPG axis interact and exacerbate the symptoms of neuroendocrine disorders in PCOS. Additionally, neurotransmitters such as GABA, dopamine, beta-endorphin, and norepinephrine are involved in the regulation of these two axes.

Some studies have found that intracerebroventricular injection of gastric inhibitory polypeptide significantly decreases serum LH levels and pulse rate in rats (60). The increase in PYY and gastric inhibitory polypeptide affects the secretion of sex hormones from the pituitary gland and hypothalamus through the gut-brain axis, leading to the hypothesis that brain-gut mediators may be involved in the occurrence and development of PCOS by influencing the HPG axis and HPA axis (30). Currently, there are fewer studies on the relationship between the brain-gut axis and GM, and the relationship between the brain-gut axis serum mediator levels and PCOS remains controversial in human studies. Additionally, the specific mechanism by which brain-gut mediators act on the central endocrine system is yet to be fully understood.

## 4 Gut microbiota-based therapies

### 4.1 Diet-targeted intervention

A systematic review and meta-analysis of 39 471 women showed that women with PCOS have a lower overall diet quality, poorer dietary intakes (higher cholesterol, lower magnesium and zinc), and lower total physical activity (61). Diet-targeted intervention should be formulated according to individual patient needs and goals, covering multiple directions such as fertility regulation, menstrual management, weight loss, or hyperandrogen symptom control (62), including the Mediterranean Diet and Nutraceutical Supplementation. Studies have shown that metabolic alterations in PCOS are closely related to the vitamin D receptor (VDR) polymorphisms such as iApa-I, Taq-I, Cdx2, and Fok-I. Variants in the VDR gene and VitD3 levels can impact the clinical features of PCOS, with VDR insufficiency or deficiency being a risk factor for PCOS pathogenesis. Vitamin D significantly improves glucose metabolism by increasing insulin production, enhancing IR expression, and reducing pro-inflammatory cytokines. The effects of vitamin D on metabolic and reproductive dysfunction associated with PCOS may be mediated through its overall effect on IR. Vitamin D supplementation has been shown to improve menstrual cycles, increase folliculogenesis, and reduce blood testosterone levels in patients with PCOS, all of which positively affect fertility (63). Therefore, vitamin D is an effective treatment for PCOS (64).

### 4.2 Probiotics and prebiotics

Probiotics are live microorganisms that, when used in sufficient amounts, can improve GM health by antagonizing the growth of pathogenic microorganisms and reducing leaky gut and inflammation. They are substrates selectively utilized by host microorganisms with health benefits (65). A systematic analysis of randomized controlled trials involving overweight/obese Iranian females (aged 15-48 years) demonstrated that probiotic and Prebiotics supplementation significantly ameliorates metabolic and endocrine abnormalities: Insulin sensitivity: Marked reductions in Homeostasis Model Assessment of Insulin Resistance (HOMA-IR) index, FBG, and fasting insulin levels were observed (all P<0.05). Lipid metabolism regulation: Decreased levels of low-density lipoprotein cholesterol and triglycerides, accompanied by elevated high-density lipoprotein cholesterol. Sex hormone homeostasis: Increased serum sex hormone-binding globulin levels coupled with reduced total testosterone concentrations, indicative of attenuated hyperandrogenemia (66). In terms of blood glucose regulation, Probiotics can increase the production of SCFA to maintain the integrity of the intestinal barrier or regulate the immune response (67), activate G protein-coupled receptors, and promote the release of peptides such as GLP-1 and PYY to lower blood glucose (68). Probiotics can reduce proinflammatory cytokines such as TNF-α and interleukin-6, enhance the intestinal barrier, reduce lipopolysaccharide transport into the bloodstream, ameliorate the effect of chronic inflammation on insulin signaling, and improve insulin sensitivity (69). Resistant starch in prebiotics can promote the proliferation of butyric acid-producing bacteria, such as *Faecalibister prausnitzii*, up-regulate butyric acid synthesis-related genes, enhance colonic butyrate level, and then activate host GPR41/43 receptors to improve insulin sensitivity (70). Additionally, probiotics can influence bile acid metabolism, which acts as signaling molecules in glucose and lipid metabolism through receptors like FXR and TGR5 (71). In terms of hormone regulation, probiotics affect sex hormone levels by regulating intestinal flora and its metabolites. For example, strains such as *Lactobacillus* and *Bifidobacterium* may directly reduce testosterone levels and improve the balance of LH and FSH secretion by regulating the gut-brain axis or directly participating in hormone metabolism (72). Usman, T. O. et al. showed that acetate (SCFAs) is able to prevent the AR-dependent effects and suppress testosterone level in testosterone-exposed pregnant rats, and an alternative receptor, the orphaned G-protein coupled receptor, suppressed testosterone level in testosterone-exposed pregnant women rats (73). In terms of lipid metabolism, probiotics can significantly reduce triglycerides, total cholesterol, and low-density lipoprotein cholesterol and increase high-density lipoprotein cholesterol, effectively improving lipid disorders (74). Studies have also found that its antioxidant effect can further reduce oxidative stress damage in patients with PCOS by increasing total glutathione and total antioxidant capacity (69). A number of clinical studies have verified the above mechanism, and the results show that supplementation of probiotics and synbiotics can significantly reduce FBG, insulin levels, and HOMA-IR in PCOS patients (69). At the same time, it was observed that testosterone levels decreased and sex hormone-binding globulin levels increased, accompanied by the improvement of hirsutism and irregular menstruation (40). Supplementation with prebiotics such as RS or FOS significantly reduced HOMA-IR and free testosterone levels in PCOS patients (75); metagenomic analysis further confirmed the functional gene remodeling of the microbiota (76). These studies provide evidence for the application of probiotics and prebiotics in the regulation of hormone and lipid metabolism. Kaur I et al. showed that *Lactobacillus acidophilus* UBLA-34, L. *rhamnosus* UBLR-58, *L. reuteri* UBLRu-87, *L. plantarum* UBLP-40, *L. casei* UBLC-42, *L. fermentum* UBLF-31, *Bifidobacterium bifidum* UMBB-55, and fructo-oligosaccharides can significantly improve the clinical symptoms of PCOS (77). Prebiotics such as FOS are also notable (69).

### 4.3 Dietary flaxseed oil

FO is a functional food rich in plant-derived α-linolenic acid-3 polyunsaturated fatty acids, which has been shown to be beneficial in chronic metabolic diseases. Ting Wang et al. demonstrated that FO intervention significantly modulated the composition of the intestinal and vaginal microbiota in rats (78). Plasma lipopolysaccharide levels decreased, while short-chain fatty acids (acetic, propionic, butyric, and valeric acids) increased after fish oil was added to their feed. This analysis supports a close relationship between sex steroid hormones, inflammation, and gut/vaginal microbiota, suggesting that dietary addition of FO can help improve PCOS symptoms by modulating the sex steroid hormone-microbe-inflammation axis in rats (78).

### 4.4 Polyphenols

Polyphenols can alter the composition of GM, promoting the growth of beneficial taxa and inhibiting the growth of harmful taxa. Most sources of dietary polyphenols do not inhibit the growth of lactic acid bacteria; instead, they may stimulate the growth of certain lactic acid bacteria. Additionally, it has been found that GM can convert polyphenols into bioactive substances that affect intestinal and physical health. Polyphenols in the diet may modulate the enzymatic activity of bacterial metabolites and reduce bacterial pathogenicity (79).

## 5 Conclusions

PCOS is an endocrine disorder prevalent among women of childbearing age. Recent research increasingly suggests that GM plays a significant role in the pathogenesis of PCOS. Studies have indicated that there is a decline in the diversity and a shift in the composition of the GM in women with PCOS. Dysfunctional GM can disrupt normal receptor function and substance regulation mechanisms by compromising the intestinal mucosal barrier, impairing normal intestinal metabolism, and activating immune responses. These disruptions include glucose metabolic disorders characterized by IR, sexual hormone metabolic disorders dominated by hyperandrogenism, abnormal bile acid metabolic pathways, IL-22 mediated intestinal immune dysfunction, and dysregulation of the brain-gut axis. The occurrence of these metabolic disorders exacerbates the progression of PCOS.

However, the specific pathways through which GM influences metabolic processes remain largely unclear. Especially since GM are susceptible to interference from various external factors, it is necessary in the future to explore the impact of different dietary habits, regional living disparities, and varying genetic susceptibilities on changes in GM of patients with PCOS. Meanwhile, studying the changes in specific strains and their mechanisms of action is crucial for advancing treatment strategies based on GM.
